# Scintigraphic Evaluation of Thyroid Pyramidal Lobe

**DOI:** 10.4274/Mirt.09719

**Published:** 2013-08-01

**Authors:** Arzu Cengiz, Hatice Şakı, Yakup Yürekli

**Affiliations:** 1 Adnan Menderes University Medical School, Department of Nuclear Medicine, Aydın, Turkey

**Keywords:** Thyroid gland, scintigraphy, thyroidectomy

## Abstract

**Objective:** The aim of this study is to investigate the presence of pyramidal lobe in thyroid scintigraphy and to compare the presence of pyramidal lobe in different thyroid pathologies between genders.

**Methods:** Images of 866 patients (663 female, 203 male) with ages ranging from 8 to 85 were evaluated retrospectively. Presence of pyramidal lobe and its location were established in images. Patients were divided into groups in terms of gender, presence of nodular/diffuse goiter, thyroid function test results and rate of the presence of pyramidal lobe and whether a significant difference existed between the groups were calculated.

**Results:** Of the 866 patients, 156 (18%) had pyramidal lobe observed in scintigraphy. Hundred and 26 (81%) of patients observed to have pyramidal lobe were female and 30 (19%) were male. Pyramidal lob stemmed from the left lobe in 76 (48%) patients, right lobe in 61 (40%) patients, and isthmus in 19 (12%) patients. Pyramidal lobe visualization rate was 18% for euthyroidism and hyperthyroidism, it was found as 15% for hypothyroidism. The rate of pyramidal lobe visualization was 13% in nodular goiter patients, 43% in diffuse goiter patients, and 20% in patients whose scintigraphy showed normal thyroid glands. In the statistical evaluation, rate of pyramidal lobe visualization in diffuse goiter patients was found to be significantly higher compared to other patients (p<0.001).

**Conclusion:** Preoperative imaging of pyramidal lobe especially in patients requiring total thyroidectomy would decrease relapses that may occur later and thus facilitate the treatment and monitoring of patients.

**Conflict of interest:**None declared.

## INTRODUCTION

Pyramidal lobe is an embryonic remnant of thyroglossal duct and is observed as a structure going upwards from right or left thyroid lobe or isthmus. In literature, prevalence of pyramidal lobe was reported as ranging between 15 to 75% (1). In patients that would undergo thyroid surgery, determining pyramidal lobe preoperatively has importance in three aspects: 1. Recurrent hyperthyroidism may develop due to insufficient resection in patients with Graves’ disease who undergo total thyroidectomy 2. Pyramidal lobe may be a source of tumor or metastasis for some tumors that can be multifocal, especially in papillary cancers. 3. In malignant thyroid cancer patients who undergo radioactive iodine treatment (RAI) following total thyroidectomy, it prevents the increase of TSH and thus decreases the possibility to benefit from the treatment. (1,2). Detection of pyramidal lobe by thyroid scintigraphy was reported to be ranging from 4.2% to 40% (3,4,5). The aim of this study is to investigate the presence of pyramidal lobe in thyroid scintigraphy and to compare the presence of pyramidal lobe between genders in different thyroid pathologies.

## MATERIALS AND METHODS

Images of 1026 patients whose thyroid scintigraphies were conducted at our Nuclear Medicine Clinic between January 2010 and March 2011 were assessed retrospectively. Patients who underwent thyroid operation, patients with congenital hypothyroidism and patients with decreased uptake due to significant suppression, and whose clinical results could not be obtained were excluded and 866 patients (663 female, 203 male) were included in the study. Patients were between 8 and 85 year-old (mean 51.3).

Thyroid scintigraphy images were acquired 20 minutes after the intravenous injection of 5 mCi Tc 99m pertechnetate, as 100 Kcounts from anterior in supine position. Siemens e.cam Single Head? Gamma camera and pinhole collimator was used for imaging. Patients were given at least 200 ml of water to drink before imaging in order to prevent possible esophageal artifact. In pre-imaging period, medical history, physical examination findings, ultrasonography reports, and thyroid function tests of patients within last month were recorded. Before scintigraphic imaging, thyroid hormone preparations were withheld three weeks ago and antithyroid medications were withheld three days ago. Patients were drug free when their thyroid functions tests were evaluated. Thyroid scintigraphic images were evaluated by two experienced nuclear medicine physicians. There was no discordance between the observers in the assessment of scans. Images of each patient were divided as nodular or diffuse goiter or normal scintigraphic appearance. Nodule activity and location in presence of nodular goiter were noted. Presence and location of pyramidal lobe were established. Patients were divided into groups as euthyroid, hypothyroid, and hyperthyroid based on thyroid function test results. Patients were individually grouped in accordance with gender, presence of nodular/diffuse goiter, and thyroid function test results, and rate of the presence of pyramidal lobe and whether there was a significant difference between the groups were calculated. 

**Statistical Assessment**

Pearson’s chi-square test was used for the assessment of pyramidal lobe between (genders) in different thyroid pathologies. p<0.05 was accepted to be statistically significant.

## RESULTS

In this study, pyramidal lobe was observed in 156 scintigraphies (18%) of the total of 866 patients. 126 (81%) of the patients observed to have pyramidal lobe were female and 30 (19%) were male. The difference in pyramidal lobe visulization between genders was found to be statistically significant (p<0.005). When an assessment was carried out on the entire patients, pyramidal lobe prevalence was 15% (30/203) among male patients and 19% (126/663) among female patients.

**Figure 1:**
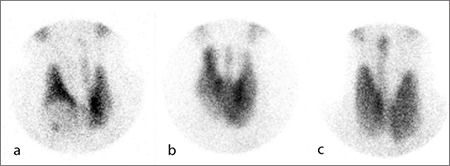
Pyramidal lobe is observed to originate from (a) left lobe, (b) isthmus, and (c) right lobe in thyroid scintigraphy

Pyramidal lobe originated from the left lobe in 76 (48%) patients, right lobe in 61 (40%) patients, and isthmus in 19 (12%) patients (Figure 1). 

When evaluated according to thyroid function tests, 418 patients were observed to have euthyroidism, 367 patients had hyperthyroidism, and 81 patients had hypothyroidism. While the pyramidal lobe visualization rate in the patient groups was 18% for euthyroidism and hyperthyroidism, it was found to be 15% for hypothyroidism (Table 1). The assessment did not show a statistically significant difference between the groups (p=0.732). In patients with pyramidal lobe, 49% were euthyroid, 43% were hyperthyroid and 8% were hypothyroid. In patients without pyramidal lobe, 48% were euthyroid, 42% were hyperthyroid and 10% were hypothyroid. 

When patients were divided as nodular/diffuse goiter or normal thyroid glands according to their scintigraphic images, 694 (80%) of the patients had nodular goiter, 142 (16%) had diffuse goiter, and 30 (4%) had normal thyroid gland findings. Pyramidal lobe prevalence was 13% in nodular goiter patients, 43% in diffuse goiter patients, and 20% in individuals with normal thyroid gland in scintigraphy. (Table 2). In the statistical assessment, pyramidal lobe visualization rate in patients with diffuse goiter was found to be significantly higher compared to other patients (p<0.005). Patients with pyramidal lobe evaluated according to normal, nodular, diffuse goiter and thyroid function tests are given in Table 3.

In the assessment of 694 nodular goiter patients, pyramidal lobe visualization rate in patients with hyperactive nodule was 11%, whereas it was 14% in patients with hypoactive and/or normoactive nodular goiter (p=0.263). 

When patients were grouped according to their age, prevalence of pyramidal lobe was higher in the younger age group and the rate was decreasing with older age. Pyramidal lobe visualization between age groups was statistically significant (p<0.005) (Table 4).

## DISCUSSION

There is differing information in literature on the prevalence of the pyramidal lobe which is an embryonic remnant of thyroglossal duct. It is reported as ranging from 15% to 75% in anatomy books (6,7,8). In a cadaver study, Braun et al. reported the pyramidal lobe prevalence as 55%, while in another cadaver study, the reported rate was 28.9% (1).

Thyroid cells found in pyramidal lobe are not active in general; however, they can become active after excision of the functioning thyroid tissue. In that case, relapsing hyperthyroidism may develop in patients that undergo total thyroidectomy due to Graves’ disease. Other than this, cases of thyroid cancer detected in pyramidal lobe have also been reported (2,10). Pyramidal lobe retained in thyroidectomized patients who are planned to undergo postoperative radioactive iodine treatment would cause TSH not to increase enough hence it may give rise to reduction in the efficiency of treatment. Due to such reasons, it is important to be preoperatively aware of the presence and location of pyramidal lobe in patients who will undergo thyroid operation. Detection of pyramidal lobe by radionuclide methods were reported at a rate ranging from 4.2 to 40% (3,4,5). It is lower compared to anatomic and surgical methods, and this is attributed to the fact that pyramidal lobe has quite a thin structure and it is not functionally active in patients who did not undergo thyroid operation (1,11). Esophageal activity may be confused with the pyramidal lobe on the scintigraphic images. Patients must drink water shortly before imaging in order to prevent possible esophageal artifact. Additionally, anterior oblique images may be helpful for differential diagnosis. Pyramidal tissue is located in the anterior of the gland whereas esophageal activity is located posteriorly (12). 

Pyramidal lobe prevalence was found to be 18% in our study. In literature, prevalence of pyramidal lobe differ greatly between genders. There are articles that suggest it is prevalent among females (13) and some others note it is more frequently observed among males (14). 

In literature, there is data on the location of pyramidal lobe, being more prevalent in the left lobe (1,15,16). This may be due to the fact that thyroglossal duct is generally developed in the left caudal direction. In line with the literature, pyramidal lobe was most frequently observed in the left lobe (48%) in our study. Pyramidal lobe was present in the right lobe in 40% of the patients, and in 12% it originated from isthmus. In a study conducted by Wahl et al., pyramidal lobe was observed to have left lobe origin at 53%, right lobe origin at 39% and isthmus at 8% (16).

In thyroid scintigraphy, pyramidal lobe is observed in higher rates in patients who underwent thyroid operation or radioactive iodine treatment, patients with hyperthyroidism and large thyroid gland (4). When patients were divided into groups based on thyroid function tests, the presence of pyramidal lobe was similar (18%) among patients with hyperthyroidism and euthyroidism in our study. Pyramidal lobe was present in 15% of the hypothyroid patients. In addition to euthyroidism, hyperthyroidism and hypothyroidism rates were similar in patients with and without pyramidal lobe. In another study, pyramidal lobe was observed at a rate up to 81% among Graves’ patients and up to 70% among autoimmune thyroiditis patients, and pyramidal lobe prevalence was suggested to increase through the rise of thyroid stimulating factors in patients with latent hypothyroidism due to iodine deficiency and in Graves’ disease patients (16). The fact that our country is located endemically in iodine-deficient region may be an explanatory factor in the prevalence of pyramidal lobe among euthyroidism and hypothyroidism patients at rates similar to hyperthyroidism patients. In addition, we showed in our study that the prevalence of pyramidal lobe in patients with diffuse goiter was significantly higher compared to patients with nodular goiter and normal scintigraphic findings. The fact that a vast majority of diffuse goiter patients in our study comprised of Graves and autoimmune thyroiditis patients, this may have an effect on higher rates of pyramidal lobe visualization in this group. In a scintigraphic study conducted by Levy et al., prevalence of pyramidal lobe was found to be 43% in diffuse toxic goiter, 17% in normal thyroid glands, 11% in hypoactive nodular goiter, and 10% in solitary functioning nodules (11). In our study, too, pyramidal lobe rates were found to be quite similar to the aforementioned study. Although prevalence of pyramidal lobe in patients with hyperactive nodule was lower than patients with hypoactive and/or normoactive nodules (11%/14%), the difference was not statistically significant. In hyperactive nodular goiter, failure to establish pyramidal lobe may be possible due to suppression in peripheral tissue. In our study, prevalence of pyramidal lobe was higher in the younger age group and the rate was decreasing with older age. It can be explained by the higher incidence of Graves disease in young people and rate of pyramidal lobe visualization is higher in Graves disease (16).

In conclusion, the prevalence of pyramidal lobe visualization in thyroid scintigraphy was 18% in this retrospective study. Imaging of pyramidal lobe preoperatively especially in patients requiring total thyroidectomy would facilitate the treatment and monitoring of the patient by decreasing relapses that may occur at a later time. Thyroid scintigraphy can easily image the pyramidal lobe and should be conducted in every patient before the operation, and if pyramidal lobe is visualized, its location should be reported.

## Figures and Tables

**Table 1:**
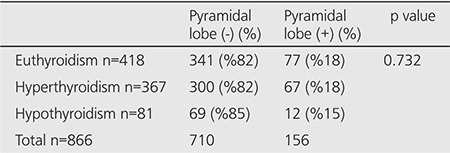
Grouping of patients according to the visualization of the pyramidal lobe and thyroid function tests

**Tablo 2:**
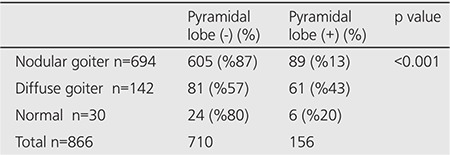
Distribution of patients according to the visualization of the pyramidal lobe n the presence of diffuse goiter, nodular goiter, and normal thyroid scintigraphy

**Table 3:**
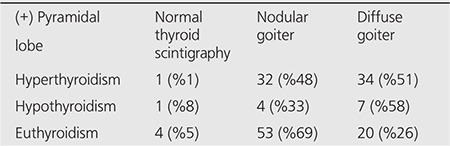
Patients with pyramidal lobe groupedaccording to scintigraphic findings and functional status of the thyroid

**Table 4:**
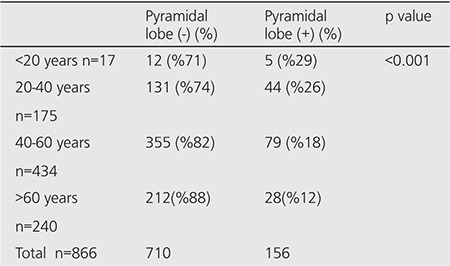
Age groups with or without pyramidal lobe visualized in scintigraphy
